# Supplementary material for: Modelling opinion dynamics under the impact of influencer and media strategies
Source: Sci Rep. 2023 Nov 8;13:19375. doi: 10.1038/s41598-023-46187-9 (PMC10632524; doi:10.1038/s41598-023-46187-9)
Supplement: Supplementary file 1 — Supplementary Information. [file 41598_2023_46187_MOESM1_ESM.pdf]

Supplementary Information for

Modelling opinion dynamics under the impact of influencer and media strategies

Luzie Helfmann, Nataša Djurdjevac Conrad, Philipp Lorenz-Spreen and Christof Schütte

Derivation of the partial mean-field model

In the following, we will outline the derivation of the partial mean-field model for the limiting opinion dynamics of infinitely many individuals, while keeping the number of media and influencers fixed. Related mean-field PDEs have already been derived in the context of other opinion models [11, 6, 7].

Assuming constant influencer-follower connections

For the derivation, we will assume that the network between individuals is fully-connected, i.e. that $A_{ij} = 1$ for all i, j, and that each individual follows exactly one medium and one influencer. Further in this first part, we will suppose that individuals do not change the influencer they are following, i.e. the matrix of influencer-follower relations remains constant $C(t) \equiv C$. In a next step we will relax this assumption. For the purpose of this derivation, we add the superscript (N) to the model quantities whenever the number of individuals is still finite and we have not yet taken the limit.

Since there are M media and L influencers in the system, we have $M \times L$ different types of individuals depending on which combination of a medium and an influencer a particular individual is following. Since the network between individuals is fully-connected, individuals that follow the same influencer and medium can be considered as identical (exchangeable). For that reason, we can switch from a description in terms of labeled individuals to a description in terms of empirical distributions. Let us define the empirical distribution of individuals that follow medium m and influencer l by

$$\rho_{m,l}^{(N)}(x,t) := \frac{1}{N} \sum_{\substack{i:B_{im}^{(N)}=1,\\ C_{il}^{(N)}(t)=1}} \delta(x - x_i^{(N)}(t)) \tag{1}$$

and the proportion of these individuals compared to the total number of individuals in the system, N, by

$$n_{m,l}^{(N)}(t) := \int_D \rho_{m,l}^{(N)}(x,t)dx.$$

The proportion $n_{m,l}^{(N)}(t)$ is constant in time for now, since influencer-follower relations cannot be changed. Further, we denote the total empirical distribution of individuals that follow any influencer or medium by

$$\rho^{(N)}(x,t) := \frac{1}{N} \sum_{i=1}^{N} \delta(x - x_i^{(N)}(t)) = \sum_{m=1}^{M} \sum_{l=1}^{L} \rho_{m,l}^{(N)}(x,t) \tag{2}$$

with $\int_D \rho^{(N)}(x,t)dx = \sum_{m,l} n_{m,l}^{(N)}(t) = 1$.

The opinion dynamics of individuals $i = 1, \dots, N$ are given by

$$dx_i^{(N)}(t) = F_i(\boldsymbol{x}^{(N)}, \boldsymbol{y}^{(N)}, \boldsymbol{z}^{(N)})dt + \sigma dW_i(t). \tag{3}$$

With the definition of the empirical distribution in (2), we can rewrite the interaction force F_i as

$$F_i\left(\boldsymbol{x}^{(N)}, \boldsymbol{y}^{(N)}, \boldsymbol{z}^{(N)}\right) = a\,\frac{\int_D \rho^{(N)}(x,t)\,\phi\left(\|x - x_i^{(N)}(t)\|\right)\left(x - x_i^{(N)}(t)\right)\,dx}{\int_D \rho^{(N)}(x,t)\,\phi\left(\|x - x_i^{(N)}(t)\|\right)\,dx} + b\left(y_m^{(N)}(t) - x_i^{(N)}(t)\right) + c\left(z_l^{(N)}(t) - x_i^{(N)}(t)\right)$$

whenever the agent i follows medium m and influencer l. Thus the SDE in (3) depends no longer on the distinct opinions of other individuals, but instead on the empirical distribution $\rho^{(N)}$.

We are now interested in the limit $N \to \infty$. We assume that as N grows, the number of individuals that follow a certain medium m and a certain influencer l grows like $Nn_{m,l}^{(N)}(t)$ with $n_{m,l}^{(N)}(t)$ independently of N. It can then be expected [10, 1] that as $N \to \infty$, any individual that follows a certain medium m and influencer l has an i.i.d. distributed opinion $\bar{x}_{m,l}$ that satisfies the SDE

$$d\bar{x}_{m,l}(t) = \mathcal{F}(\bar{x}_{m,l}, y_m, z_l, \rho)dt + \sigma dW(t) \tag{4}$$

with interaction force

$$\mathcal{F}(\bar{x}_{m,l}, y_m, z_l, \rho) = a\,\frac{\int_D \rho(x,t)\,\phi\left(\|x - \bar{x}_{m,l}\|\right)(x - \bar{x}_{m,l})\,dx}{\int_D \rho(x,t)\,\phi\left(\|x - \bar{x}_{m,l}\|\right)\,dx} + b\,\left(y_m(t) - \bar{x}_{m,l}\right) + c\,\left(z_l(t) - \bar{x}_{m,l}\right).$$

The opinion process $\bar{x}_{m,l}$ from (4) depends on the density ρ which can be interpreted as the limit of $\rho^{(N)}$ when $N \to \infty$. More precisely, denoting by $\mu_{m,l}(x,t)$ the probability density of an individuals' opinion with medium m and influencer l at time t, $\rho_{m,l}(x,t) := n_{m,l}(t)\mu_{m,l}(x,t)$ denotes the probability density scaled by the proportion of these individuals in the system, and $\rho(x,t) = \sum_{m,l}\rho_{m,l}(x,t)$ denotes the probability density of any individual in the system. Stochastic processes of the form (4) are also called *McKean-Vlasov processes*. Each scaled density $\rho_{m,l}$ fulfils a *McKean Vlasov-type PDE*

$$\partial_t \rho_{m,l}(x,t) \quad = \quad \frac{1}{2}\sigma^2 \Delta \rho_{m,l}(x,t) - \nabla \cdot \left(\rho_{m,l}(x,t)\,\mathcal{F}(x, y_m, z_l, \rho)\right) \tag{5}$$

and we can also interpret each PDE for a fixed m,l as describing the density of infinitely many copies of individuals that follow medium m and influencer l. There is also a growing body of literature that considers an intermediate level of many but not infinitely many individuals, whose empirical distribution in the opinion space can approximately be described by a stochastic partial differential equation [3, 9, 8, 4].

Allowing influencer-follower connections to change

We now additionally allow individuals to switch the influencer in time. In the ABM each individual i can change the influencer to l at the rate $\Lambda_m^{\to l^{(N)}}(x,t)$ where m is the medium of individual i and x its current opinion. In the PDE we want these changes from influencer l' to l to correspond to mass flowing from $\rho_{m,l'}$ to $\rho_{m,l}$. We will in the following derive the corresponding terms that have to be added to the PDE (5) and are sometimes also called reaction terms.

The total number of individuals that follow medium m and influencer l at time t in the ABM is given by $Y_{m,l}^{(N)}(t) := N\,n_{m,l}^{(N)}(t)$ and can be considered a jump process that only changes by $+1$ when an individual changes its influencer to l or by -1 when an individual changes its influencer away from l. The rate of *any* individual changing from (m,l') to (m,l) is given by the sum of the individual change rates as follows

$$\alpha_m^{l' \to l^{(N)}}(t) = \sum_{\substack{i:\, B_{im}^{(N)}=1,\\ C_{il'}^{(N)}(t)=1}} \Lambda_m^{\to l^{(N)}}(x_i,t) = N\int_D \Lambda_m^{\to l^{(N)}}(x,t)\,\rho_{m,l'}^{(N)}(x,t)\,dx.$$

With this, we can write down the evolution of the jump process $Y_{m,l}^{(N)}(t)$ as

$$Y_{m,l}^{(N)}(t) = Y_{m,l}^{(N)}(0) + \sum_{l' \neq l}\left(\mathcal{P}_m^{l' \to l}\left(\int_0^t \alpha_m^{l' \to l^{(N)}}(t')\,dt'\right) - \mathcal{P}_m^{l \to l'}\left(\int_0^t \alpha_m^{l \to l'^{(N)}}(t')\,dt'\right)\right)$$

with unit-rate Poisson processes $\mathcal{P}_m^{l\to l'}$.

In the limit $N \to \infty$, the rates become large and we can replace the Poisson processes by their mean to get the *reaction rate equation* [12] written in terms of the limiting proportions $n_{m,l}(t)$

$$\frac{dn_{m,l}}{dt}(t) = \sum_{l'\neq l} \left(n_{m,l'}(t)\, r_m^{l'\to l}(t) - n_{m,l}(t)\, r_m^{l\to l'}(t) \right) \tag{6}$$

with the spatially-averaged rates in the limit

$$r_m^{l'\to l}(t) = \frac{1}{n_{m,l'}(t)} \int_D \Lambda_m^{\to l}(x,t)\, \rho_{m,l'}(x,t)\, dx$$

and the limiting individual change rates

$$\Lambda_m^{\to l}(x,t) = \eta\, \psi\left(\|z_l - x\|\right)\, r\left(\frac{n_{m,l}(t)}{\sum_{m'} n_{m',l}(t)} \right).$$

The ODE (6) can be spatially extended to the following term [9, 8]

$$\partial_t \rho_{m,l}(x,t) = \sum_{l'\neq l} -\Lambda_m^{\to l'}(x,t)\, \rho_{m,l}(x,t) + \Lambda_m^{\to l}(x,t)\, \rho_{m,l'}(x,t), \tag{7}$$

such that the complete PDE describing opinion changes and influencer changes is given by the sum of (5) and (7),

$$\begin{aligned} \partial_t \rho_{m,l}(x,t) =& \frac{1}{2}\sigma^2 \Delta \rho_{m,l}(x,t) - \nabla \cdot \left(\rho_{m,l}(x,t)\, \mathcal{F}(x, y_m, z_l, \rho) \right) \\ &+ \sum_{l'\neq l} \left(-\Lambda_m^{\to l'}(x,t)\, \rho_{m,l}(x,t) + \Lambda_m^{\to l}(x,t)\, \rho_{m,l'}(x,t) \right). \end{aligned} \tag{8}$$

Limiting SDE dynamics of influencers and media

The limiting dynamics of media and influencers follow by considering the limiting average position of followers of medium m

$$\begin{aligned} \tilde{x}_m^{(N)}(t) &= \frac{1}{\sum_{k=1}^N B_{km}^{(N)}} \sum_{i=1}^N B_{im}^{(N)} x_i^{(N)}(t) \\ &= \frac{\sum_{l=1}^L \int_D x\, \rho_{m,l}^{(N)}(x,t)\, dx}{\sum_{l=1}^L n_{m,l}^{(N)}(t)} \\ &\to \frac{\sum_{l=1}^L \int_D x\, \rho_{m,l}(x,t)\, dx}{\sum_{l=1}^L n_{m,l}(t)} =: \tilde{x}_m(t) \end{aligned}$$

and of influencer l

$$\begin{aligned} \hat{x}_l^{(N)}(t) &= \frac{1}{\sum_{k=1}^N C_{kl}^{(N)}(t)} \sum_{i=1}^N C_{il}^{(N)}(t)\, x_i^{(N)}(t) \\ &= \frac{\sum_{m=1}^M \int_D x\, \rho_{m,l}^{(N)}(x,t)\, dx}{\sum_{m=1}^M n_{m,l}^{(N)}(t)} \\ &\to \frac{\sum_{m=1}^M \int_D x\, \rho_{m,l}(x,t)\, dx}{\sum_{m=1}^M n_{m,l}(t)} =: \hat{x}_l(t). \end{aligned}$$

No-flux boundary conditions

We are interested in finding boundary conditions such that the total number of individuals in the system remains constant in time, i.e., we want

$$\int_D \partial_t \rho(x,t) dx = 0$$

for all t. We can equivalently phrase this using the PDE equation (8) and the divergence theorem as

$$\begin{aligned} 0 &= \int_D \partial_t \rho(x,t)\,dx \\ &= \sum_{m,l} \int_D \frac{1}{2}\sigma^2 \Delta \rho_{m,l}(x,t) - \nabla \cdot (\rho_{m,l}(x,t)\,\mathcal{F}(x,y_m,z_l,\rho))\,dx \\ &= \sum_{m,l} \int_{dD} \left(\frac{1}{2}\sigma^2 \nabla \rho_{m,l}(x,t) - \rho_{m,l}(x,t)\,\mathcal{F}(x,y_m,z_l,\rho) \right) \cdot n\,dx \end{aligned}$$

where dD is the boundary of D, n the unit outer normal to D. The terms for influencer changes disappeared since they only shift mass between the densities $\rho_{m,l}$ and therefore disappear when summing over m, l. A sufficient condition to ensure mass conservation is therefore to ensure that the balance equation

$$\frac{1}{2}\sigma^2 \nabla \rho_{m,l}(x,t) \cdot n = \rho_{m,l}(x,t) \mathcal{F}(x,y_m,z_l,\rho) \cdot n$$

holds everywhere on the boundary of D.

Parameter studies of the agent-based model

In this section, we will first provide some exemplary simulations of the agent-based model for different configurations of the parameters a, b and c to show the diverse dynamics of the model. And second, we will analyse the clustering of individuals when varying the interaction strength with media, b, and influencers, c.

Exemplary Simulations

We provide some example realizations of the agent-based model for different parameter settings to show the qualitatively different situations that can be modelled. In Fig. S1 we set $a = 3$, $b = c = 1$, all other parameters are kept as before. Due to the attraction force to other individuals being much stronger, individuals get attracted to the center of the domain (i.e. the average opinion of all individuals) before forming two clusters. In Fig. S2 we set $b = 3$, $a = c = 1$, thus making media agents much more influential. The strong attraction to media agents results in the quick formation of two clusters. The clusters are mixed with individuals that follow very different influencers. Most of the individuals then also change the influencer to the influencer near the medium they are close to.

Clustering dynamics for different interaction strengths

For analysing the clustering of individuals in the opinion space, we will only vary the interaction strength with media b and influencers c from 0.25 to 4.0 and keep all other parameters constant. In particular the interaction strength with individuals is given by $a = 1$. We are interested in understanding the effect of these parameters on the clustering of individuals at the time point $t = 2$ and we average the results over 10 simulations per parameter choice. Note that we have to make a choice of a time point here and we choose this as a time point where the dynamics are neither fully converged to one cluster nor close to their initial state. Also, we do not vary a because the ratios between a, b, and c determine the qualitative clustering dynamics and varying b and c is therefore sufficient. The size of these parameters only determines the time scale of the dynamics.

In Fig. S3, using the DBSCAN algorithm [5, 2], we study the number of clusters that individuals form at the chosen time point. The analysis indicates that for larger values of b (i.e. larger interaction strength with media) and smaller values of c (i.e. smaller interaction strength with influencers) individuals are on average grouped into 2 clear clusters. These clusters are positioned around the media and the two influencers at $(-1, 1)$ and $(1, 1)$, as can be observed from Figure S2. When $c > b$, we see more clusters and this number is increasing up to 4 as c is growing. This is due to the strong impact of four influencers that pull the individuals towards their positions. Note that in this scenario clusters are often not very well separated, such that depending on the choice of the clustering method and its parameters one can obtain clusters of different granularity, e.g. in the realization shown in Example 1

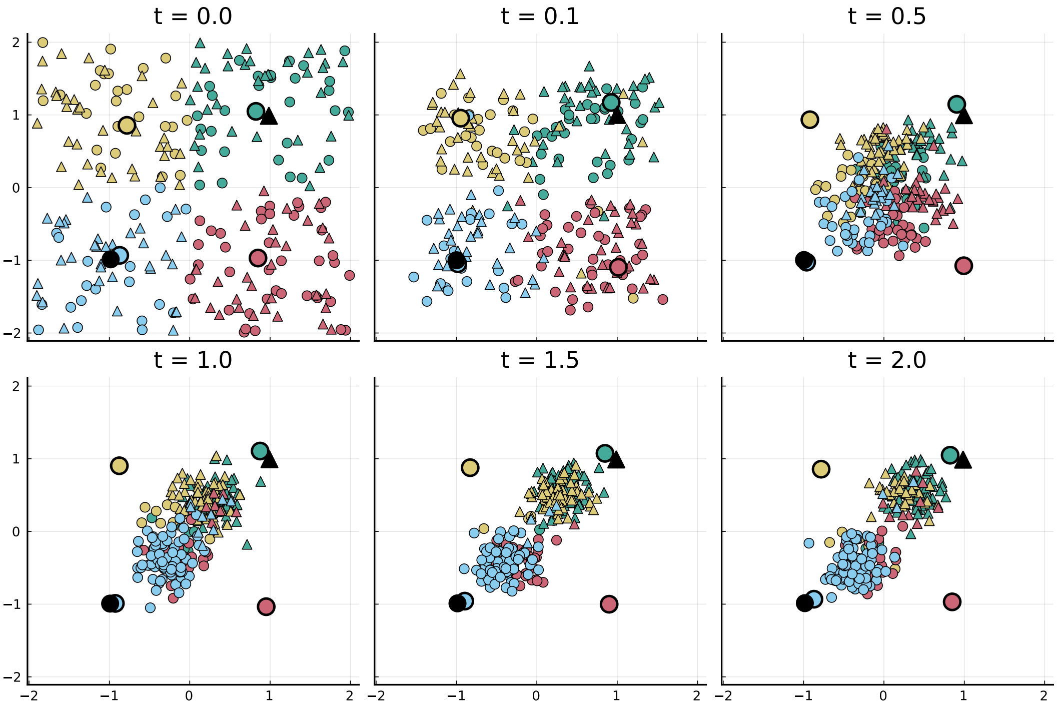

Figure S1: Realization of the ABM when setting $a = 3$, $b = c = 1$.

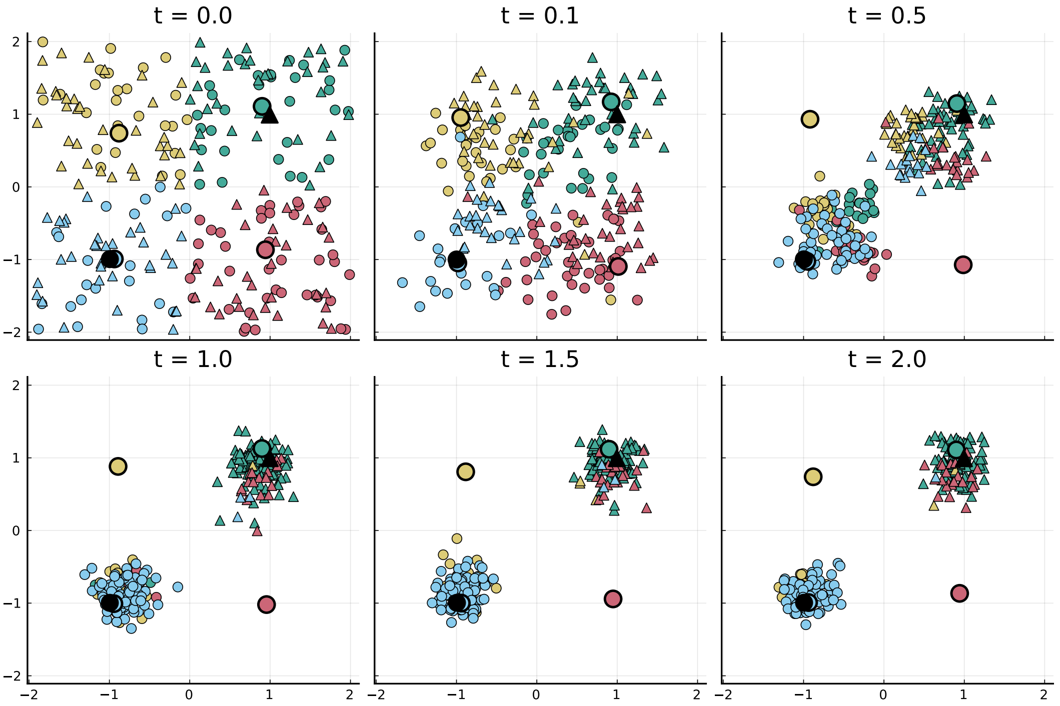

Figure S2: Realization of the ABM when choosing $b = 3$, $a = c = 1$.

in the paper. For small values of b, c, DBSCAN can only find one cluster in the opinion space. This is because the interaction strength between individuals is now the strongest and individuals get attracted to each other forming one cluster, similar as in Fig. S1.

Additionally, inspired from [11, 7], we define two order parameters $\theta^M(t)$ resp. $\theta^I(t)$ that measure

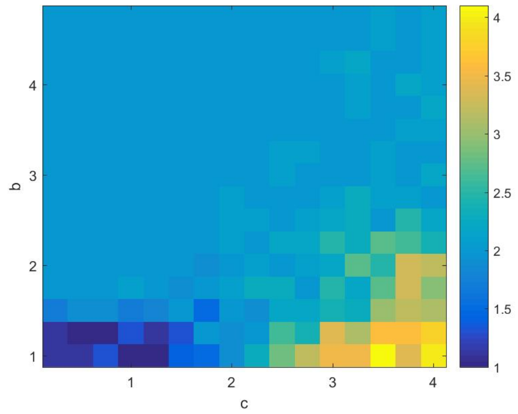

Figure S3: Average number of clusters at $t = 2.0$ for different choices of parameters b, c and 10 simulations each. Colors indicate the number of clusters that are found using DBSCAN algorithm. Other parameters are as in Example 1.

how clustered individuals are around their media resp. influencers at time t. We define them as

$$\theta^I(t) := \frac{1}{N} \sum_{\substack{i:1,\ldots,N,\\ l=1,\ldots,L}} C_{il}(t)\, \phi(|x_i - z_l|)$$

and

$$\theta^M(t) := \frac{1}{N} \sum_{\substack{i:1,\ldots,N,\\ m=1,\ldots,M}} B_{im}(t)\, \phi(|x_i - y_m|)$$

with interaction function $\phi(x) = exp(-x)$. When all individuals have exactly the same opinion as their medium (or, influencer), the order parameter is maximal and given by 1[1]. When, on the other hand, all individuals are far away from their media (or, influencers), then the interaction function of their distance is close to 0 and so are the order parameters. The order parameter thus always lies in the interval $[0, 1]$ with a larger value indicating more clustering around media resp. influencers. The order parameters at time $t = 2.0$ are shown in Fig. S4. The phase diagram of order parameters confirms the observations from the previous clustering analysis. Larger values of $\theta^M(t)$, and thus more clustering around media, appear when b is large, probably because the stronger attraction to media agents results in individuals being more densely clustered around their medium. The smallest values of $\theta^I(t)$ appear when b and c are small and thus the attraction to individuals is the strongest, probably resulting in individuals being furthest away from their influencers.

References

[1] Louis-Pierre Chaintron and Antoine Diez. Propagation of chaos: a review of models, methods and applications. *arXiv preprint arXiv:2106.14812*, 2021.

[2] Michal Daszykowski, Beata Walczak, and DL Massart. Looking for natural patterns in data: Part 1. density-based approach. *Chemometrics and Intelligent laboratory systems*, 56(2):83–92, 2001.

[3] David S Dean. Langevin equation for the density of a system of interacting Langevin processes. *Journal of Physics A: Mathematical and General*, 29(24):L613, 1996.

[4] Nataša Djurdjevac Conrad, Jonas Köppl, and Ana Djurdjevac. Feedback loops in opinion dynamics of agent-based models with multiplicative noise. *Entropy*, 24(10):1352, 2022.

[5] Martin Ester, Hans-Peter Kriegel, Jörg Sander, Xiaowei Xu, et al. A density-based algorithm for discovering clusters in large spatial databases with noise. In *kdd*, volume 96, pages 226–231, 1996.

[1] assuming that each individual is always connected to exactly one influencer and medium

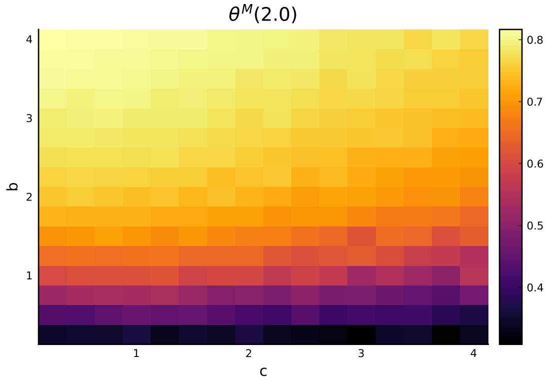

(a)

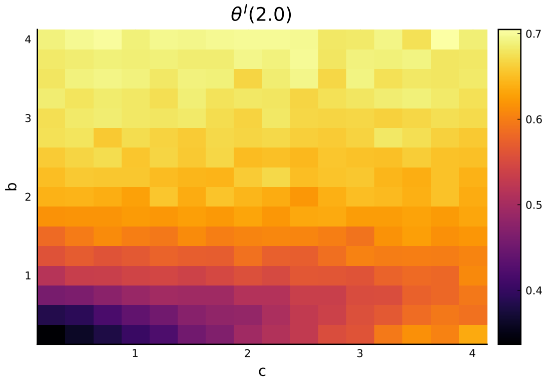

(b)

Figure S4: The order parameters for different choices of b and c, as an average over 10 simulations. Colour denotes the order parameters at the final time $t = 2.0$. Other parameters are as in Example 1.

[6] Josselin Garnier, George Papanicolaou, and Tzu-Wei Yang. Consensus convergence with stochastic effects. *Vietnam Journal of Mathematics*, 45(1):51–75, 2017.

[7] Benjamin D Goddard, Beth Gooding, H Short, and GA Pavliotis. Noisy bounded confidence models for opinion dynamics: the effect of boundary conditions on phase transitions. *IMA Journal of Applied Mathematics*, 87(1):80–110, 2022.

[8] Luzie Helfmann, Nataša Djurdjevac Conrad, Ana Djurdjevac, Stefanie Winkelmann, and Christof Schütte. From interacting agents to density-based modeling with stochastic PDEs. *Communications in Applied Mathematics and Computational Science*, 16(1):1–32, 2021.

[9] Changho Kim, Andy Nonaka, John B Bell, Alejandro L Garcia, and Aleksandar Donev. Stochastic simulation of reaction-diffusion systems: A fluctuating-hydrodynamics approach. *The Journal of chemical physics*, 146(12):124110, 2017.

[10] Alain-Sol Sznitman. Topics in propagation of chaos. In *Ecole d'été de probabilités de Saint-Flour XIX—1989*, pages 165–251. Springer, 1991.

[11] Chu Wang, Qianxiao Li, Weinan E, and Bernard Chazelle. Noisy Hegselmann-Krause systems: Phase transition and the 2R-conjecture. *Journal of Statistical Physics*, 166(5):1209–1225, 2017.

[12] Stefanie Winkelmann and Christof Schütte. *Stochastic Dynamics in Computational Biology*, volume 645. Springer, 2020.
